# Leveraging an open access platform to provide organizational value in clinical environments

**DOI:** 10.5195/jmla.2025.2086

**Published:** 2025-01-14

**Authors:** Aida Marissa Smith, Alexia Estabrook, Mary A. Hyde, Michele Matucheski, Eleanor Shanklin Truex

**Affiliations:** 1 aida.smith@ascension.org, Ascension Missouri, MO; 2 alexia.estabrook@ascension.org, Ascension Providence Hospital Michigan, MI; 3 mary.hyde@ascension.org, Ascension Florida, FL; 4 michele.matucheski@ascension.org, Ascension Wisconsin, WI; 5 eleanor.truex@ascension.org, Ascension Illinois, IL

**Keywords:** Clinical Librarians, Hospital Librarians, Nurses, Bibliographic Management Software, Organizational Value, Authorship, Collaboration

## Abstract

The Ascension Nurse Author Index is an example of how resource-limited clinical libraries can provide value to their organization by creating a database of peer-reviewed journal article publications authored by their nursing associates. In 2024, Ascension launched a database index to highlight its nurse authors, bring attention to subject matter expertise, foster collaboration among authors, and recognize impact within the profession. The index uses an open access platform, software intended for reference management with a public-facing cloud option, to minimize expenses. This unconventional use of the platform allowed us to capitalize on the software's bibliographic database management capabilities while allowing us to input institutional-specific metadata. By creative use of the open-access platform, librarians can successfully partner to create value for their organization by highlighting the work of its nurses.

The Ascension Nurse Author Index (Ascension, 2024) is an example of how resource-limited clinical libraries can provide value to their organization by creating a database of peer-reviewed journal article publications authored by their nursing associates. In 2024, Ascension launched a database index to highlight its nurse authors, bring attention to subject matter expertise, foster collaboration among authors, and recognize impact within the profession. The work was the result of a collaborative effort among Ascension librarians and nurse research scientists.

The index uses the open-access software platform Zotero, intended for reference management (Corporation for Digital Scholarship, 2024), with a public-facing cloud option to minimize expenses. This unconventional use of the platform capitalizes on the software's bibliographic database management capabilities while allowing for institutional-specific metadata for internal reporting. To populate the index, publications are captured through two primary methods. A REDCap survey is widely shared with nurses for self-reporting and a PubMed search alert is used to capture publications proactively. All identified publications are subsequently verified before inclusion. Eligible publications are easily added to the index. The platform's browser extension is used to capture bibliographic information from open-access websites, typically PubMed, with a single click. This process minimizes manual data entry, making it possible for a single librarian to manage the work of adding new publications to the index on a monthly basis.

The Ascension Nurse Author Index is making an impact across Ascension's multi-state healthcare system inclusive of approximately 48,000 nurses:

- Nurse authors are promoted by highlighting the index's availability through internal communications during Nurses Week.
- An executive-authored letter of appreciation is sent to all associate nurses authoring a newly published article included in the index.
- “Author's Row,” a feature on the system-level nursing intranet site relies on the index to identify and highlight recently published nurse authors.
- Annual and quarterly reports use the index to quantify nurse authorship within the organization.
- The value of nurse scholarship is publicized across the healthcare system.

By creative use of the open access platform, clinical librarians can successfully partner to create value for their organization by highlighting the work of its nurses. The nursing shortage (AACN, 2023; National Center for Health Workforce Analysis, 2022) combined with the concern that many hospitals are reassessing the value of library services (Harrow et al., 2019), makes it an opportune time for librarians in the clinical environment to support and showcase the work of the nurse.
